# Effect of Facial Acupuncture Stimulation: MRI-Based Masseter Muscle Volume Analysis and Questionnaire Evaluation

**DOI:** 10.1093/asjof/ojae109

**Published:** 2024-11-10

**Authors:** Mieko Ogino, Megumi Iijima, Yukinori Okada, Itsuko Okuda

## Abstract

**Background:**

Cosmetic acupuncture may reduce wrinkles, swelling, sagging, and facial asymmetry and may lift facial contours.

**Objectives:**

To investigate the impact of cosmetic acupuncture on masseter muscle volume and its implications for facial aesthetics.

**Methods:**

The authors included 10 apparently healthy adult females (average age, 50.3 ± 6.45 years) and performed acupuncture once a week for 8 weeks. MRI was conducted before acupuncture stimulation and 3 days after the treatment. The collected image data were analyzed using ZioCube software (ZioSoft Co., Ltd, Tokyo). Before and after the intervention, 2 evaluators measured the masseter muscle volume 3 times each and calculated the average value.

**Results:**

The average total volume of the left and right masseter muscles decreased from 40.73 ± 8.2 to 37.81 ± 8.57 cm^3^ after cosmetic acupuncture, a significant reduction of 2.92 ± 2.48 cm^3^ (7.37%, *P* < .05). All 10 subjective evaluation items showed significant decreases, particularly in facial sagging, contour, and asymmetry, confirming aesthetic improvements and psychological satisfaction among participants. Cosmetic acupuncture reduced masseter muscle volume, leading to noticeable aesthetic benefits.

**Conclusions:**

Acupuncture stimulation at acupoints around the masseter muscle can reduce the muscle volume because of muscle relaxation. The improvement in subjective evaluation and changes and decreases in masseter muscle shape enable the attainment of facial contour aesthetics, contributing to the evidence in support of cosmetic acupuncture.

**Level of Evidence: 4 (Therapeutic):**

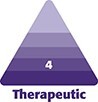

Cosmetic acupuncture, a specialized field in acupuncture therapy, is designed to reduce wrinkles, swelling, sagging, and facial asymmetry, while lifting facial contours.^1^ Researchers have shown in their studies that acupuncture can increase skin moisture, oil content, and blood flow.^2-4^ However, these researchers, through their findings, primarily focus on superficial skin changes and subjective assessments, with limited objective evaluation of subcutaneous structural changes.

As aging progresses, facial appearance changes, particularly with the jaw's shape shifting from a V shape to a trapezoidal shape,^5^ leading to increased concerns about jaw sagging and facial asymmetry. In cosmetic medicine, treatments such as Botox injections are commonly used to reduce masseter muscle volume, enhancing facial aesthetics by relaxing muscles and decreasing their activity.^6,7^

In clinical practice, cosmetic acupuncture has been reported to alleviate symptoms of temporomandibular joint disorder (TMJD) and facial asymmetry. Acupuncture therapy for TMJ has been beneficial in relieving masseter muscle tension, improving skin tone and reducing sagging.^8^ These effects suggest that the masseter muscles significantly influence facial aesthetics. Authors of preliminary MRI studies, although limited, have observed reductions in masseter muscle area and left–right asymmetry following cosmetic acupuncture,^9^ indicating its potential to enhance facial contours and reduce sagging (Video).

In this study, we aim to investigate changes in the volume of the masseter muscle through MRI-based anatomical imaging following facial acupuncture stimulation. Additionally, we conduct a survey to assess the subjective aesthetic improvements experienced by participants.

## METHODS

### Study Design

This was a nonrandomized and prospective interventional study conducted at a single institution. Both verbal and written informed consent were obtained from participants between November 25 and December 27, 2022. Patients provided written consent agreeing to the use and analysis of their data.

### Selection Criteria

Participants were females aged 40 years and older, capable of providing informed consent independently. Exclusion criteria included pregnancy or potential pregnancy, current outpatient treatment for any illness, planned facial cosmetic surgery during the study period, conditions affecting the face because of facial nerve paralysis or trauma, presence of tattoos or permanent makeup, internal metals (eg, pacemakers, cochlear implants, brain aneurysm clips, metal dental appliances, and artificial joints), and claustrophobia. Eligible participants had not received acupuncture at other facilities during the study period or within 1 month before the study. Selected participants were subjected to the Main and Subsidiary Study Items.

### Primary Investigative Items

This study was approved by the university's research ethics committee and clinical research insurance coverage. The trial was registered in the University Hospital Medical Information Network Clinical Trials Registry (trial registration number: 000049678). The study was conducted according to ethical guidelines for biomedical research involving human patients. The study was commenced on November 25, 2022, and concluded on March 1, 2023. Participants received thorough explanations, and informed consent was obtained both verbally and in writing. Minors were not included in this study.

**Figure 1.**
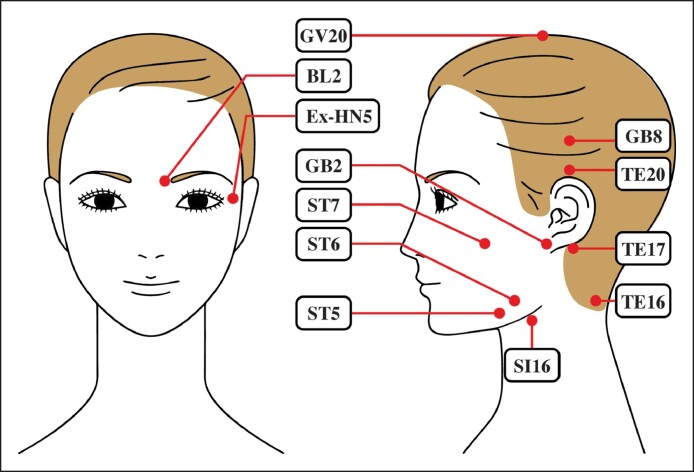
Names and locations of the acupoints. Acupuncture was performed using Seirin stainless-steel needles, with a length of 30 mm and a diameter of 0.20 mm, which were inserted to a depth of 10 mm for a duration of 15 min.

Seirin stainless-steel needles (Seirin Corporation, Shizuoka Prefecture, Japan) with a length of 30 mm and a diameter of 0.20 mm were used, with an insertion depth of 10 mm. The acupuncture was performed by an acupuncturist with 34 years of clinical experience, including 18 years of teaching experience at a vocational school's teacher training program, where a formal curriculum in cosmetic acupuncture was developed and taught. The procedure was conducted in a supine position after a 5 min resting period, for 15 min at the following facial acupuncture points (acupoints): Xiaguan (ST7), Jiache (ST6), Yifeng (TE17), Tianrong (SI16), Tianpian (TE16), Tinghui (GB2), Daying (ST5), Baihui (GV20), Zugu (GB8), Jiaosun (TE20), Zanzhu (BL2), Taiyang (Ex-HN5), and as induction points, Hege (L14) and Zusanli (ST36) on the upper and lower limbs, respectively (Figure 1).

**Figure 2.**
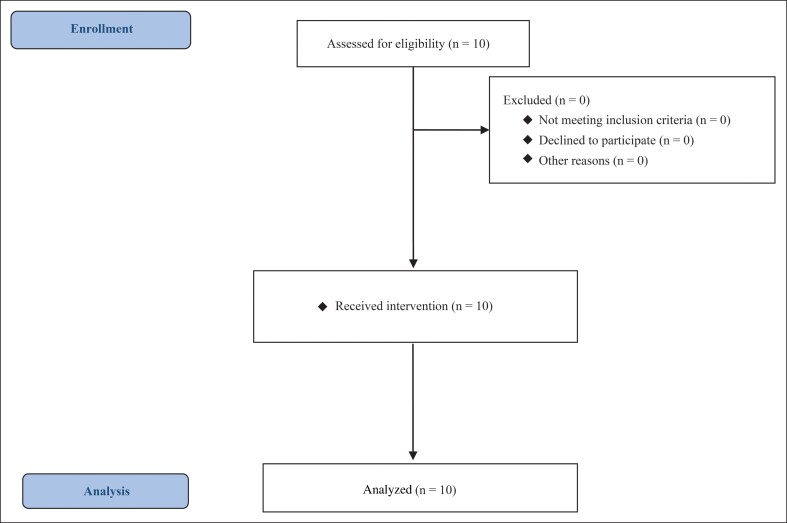
The CONSORT flow chart showing participant screening, enrollment, assignment, follow-up, and analysis stages. Acupuncture was performed weekly for 8 sessions by an acupuncturist with 30 years of experience at Kou Acupuncture Clinic.

In this study, to clarify the flow of participants, a CONSORT flow diagram is presented in Figure 2. In this flow diagram, each stage of participant screening, enrollment, assignment, follow-up, and analysis is illustrated. The intervention with acupuncture stimulation was conducted once a week for 8 sessions and was performed by an acupuncturist with 30 years of clinical experience.

The rationale for conducting the intervention once a week for 8 weeks was based on a previous study by Sato et al,^10^ wherein decreases in wrinkles and sagging and improvement in skin condition were observed. MRI was performed before the acupuncture intervention and on the third day after the completion of the 8 acupuncture sessions. We selected female participants aged 40 years and older (with a mean age of 50.3 ± 6.45 years) owing to several factors. First, the average onset age of TMJDs, which are closely associated with the masseter muscle, is reported to be 54.5 years. Second, TMJDs are more prevalent among females than among males. Additionally, considering that a higher proportion of females seek cosmetic acupuncture treatments, focusing exclusively on female participants aged 40 years and older, was deemed appropriate for this investigation.

### Equipment and Imaging

The MRI device used was the MGNETOM Lumina 3.0T (SIEMENS, Munich, Germany) with the BNHead/Neck20 coil employed. To measure muscle volume, imaging was performed to include the entire face as volume data. The protocol for sagittal imaging consisted of T1 FLASH3D, with a field of view (FOV) of 260 × 260 × 195 mm, voxel size of 1.02 × 1.02, echo time (TR) of 4.4 ms, slice thickness of 0.5 mm, and 390 slices evaluated for data acquisition. Axial images were obtained using Proton Density Weighed Imaging with Turb Spin Echo, with a voxel size of 0.5 × 0.5 mm, acquisition parameters of TR 3000 ms, voxel size 0.46 × 0.37, repetition time (TE) 20 ms, FOV 160 × 180 mm, and a slice thickness of 3.0 mm in 25 slices across 2 stacks, overlapping at boundaries.

### Measurements

**Figure 3.**
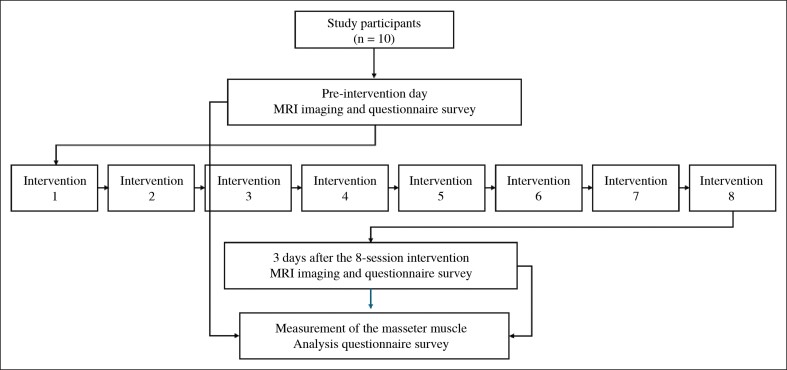
Implementation plan for cosmetic acupuncture treatment and questionnaire—MRI examination. The intervention with acupuncture stimulation was conducted once a week for a total of 8 sessions. MRI was performed before the acupuncture intervention and on the third day after the completion of the eighth acupuncture session.

The acquired image data were analyzed using ZioCube analysis software (Ziosoft Inc., Tokyo, Japan). Before and after the intervention, 2 measurers (M.O. and M.I., both with 6 years of experience in image analysis) measured the muscle volume 3 times each, totaling 6 measurements, and the mean value was calculated. To ensure accuracy, measurements were confirmed in 3 directions—axial, sagittal, and coronal imaging—covering the entire masseter muscle. Figure 3 illustrates the implementation plan for cosmetic acupuncture treatment and the questionnaires—MRI examination.

### Statistical Analysis

The primary outcome measure was the change in masseter muscle volume assessed through MRI. We conducted a paired sample *t*-test to compare the pre- and postintervention masseter muscle volume reduction and percentage change. A paired *t*-test was performed using MATLAB software (version R2023a, MathWorks Inc., Natick, MA). Statistical significance was set at *P* < .05. Additionally, seif-perceived evaluations from participant questionnaires before and after the intervention were analysed using the Wilcoxon signed-rank test, also performed with MATLAB software.

### Subsidiary Investigation

A questionnaire survey was conducted to assess participants’ self-perceived observations. The survey included physical characteristics such as weight, height, teeth grinding, and handedness, along with facial features including asymmetry, cheek sagging, jawline, nasolabial fold length, discrepancies in jawline between the left and the right sides, wrinkles around the mouth, and discrepancies in the corners of the mouth. Each item was evaluated on a 5-point Likert scale: 1, not at all concerned; 2, slightly concerned; 3, somewhat concerned; 4, concerned; and 5, very concerned. These evaluations were conducted before the intervention, 3 days after, and at 8 weeks postintervention.

## RESULTS

### Cases

Ten consecutive cases of healthy adult females aged 43 to 64 were selected. The mean age was 50.3 ± 6.45 years.

### Main Outcome Measure

**Figure 4.**
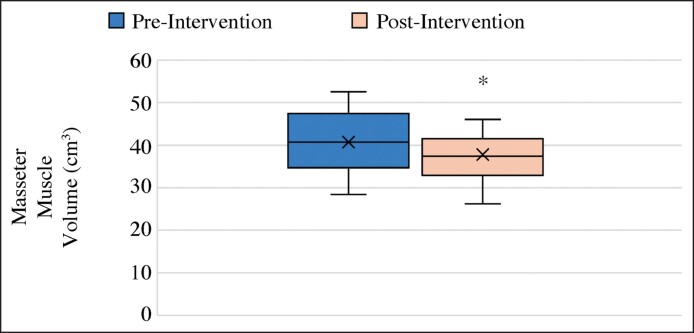
Comparison of the average volume of masseter muscles before and after cosmetic acupuncture treatment. The asterisk (*) in the box-and-whisker plot indicates a statistically significant difference.

**Figure 5.**
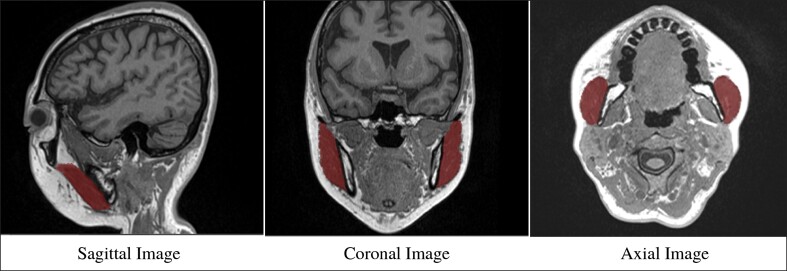
Measurement range of masseter muscles. The measurement areas on the transverse, sagittal, and coronal images are outlined in red.

Cosmetic acupuncture treatment reduced the masseter muscle volume, which participants subjectively reported as leading to aesthetic improvements. Table 1 shows the volume changes and reduction rates of the left and right masseter muscles. The mean total volume decreased from 40.73 ± 8.2 cm^3^ before treatment to 37.81 ± 8.57 cm^3^ after treatment, indicating a reduction of 2.92 ± 2.48 cm^3^ (7.37%). Figure 4 illustrates the mean volume comparison of the left and right masseter muscles before and after treatment. The reduction in masseter muscle volume was statistically significant (*P* < .05). Figure 5 shows the measurement areas in the axial, sagittal, and coronal images.

**Table 1.** Changes in the Volume and Reduction Rates of Masseter Muscles in 10 Patients After Cosmetic Acupuncture Treatment

Participant	Before (cm^3^)	After (cm^3^)	Difference (%)	Reduction rate (%)
1	52.49	55.14	2.65	−5.05
2	38.52	36.64	−1.88	4.87
3	38.74	35.18	−3.56	9.18
4	36.69	35.22	−1.47	4.01
5	46.24	40.05	−6.19	13.40
6	50.74	46.09	−4.65	9.17
7	28.39	26.21	−2.17	7.66
8	28.72	26.19	−2.53	8.80
9	44.05	39.22	−4.83	10.97
10	2.71	38.13	−4.58	10.73
Mean	40.73	37.81	−2.92	7.37
SD	8.20	8.57	2.48	5.18

SD, standard deviation.

A paired *t*-test was performed to compare the mean ± standard deviations of the masseter muscle volumes between the 2 evaluators. It showed no significant difference before (*P* = .355) and after the intervention (*P* = .625). This consistency suggests minimal variability, confirming the reliability of the measurement process. Details are given in Supplemental Table 1.

### Sample Presentation

**Figure 6.**
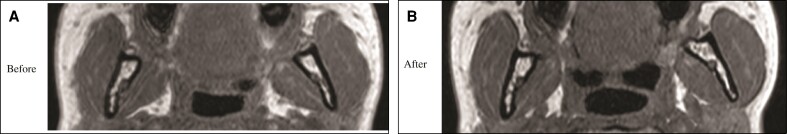
Comparison of changes in the shape of the left and right masseter muscles (A) before and (B) after cosmetic acupuncture treatment. The shape of the masseter muscle has become smoother and more defined, resulting in reduced volume. The subject in this image is a 54-year-old female.

Figure 6 shows cross-sectional images of the masseter muscles before and after cosmetic acupuncture treatment in Participant 5, a 54-year-old female. The masseter muscles decreased from 46.24 to 40.05 cm^3^, a reduction of 6.19 cm^3^ (−13.4%). Changes in the masseter muscles were observed after cosmetic acupuncture compared with before treatment.

### Secondary Investigation

**Figure 7.**
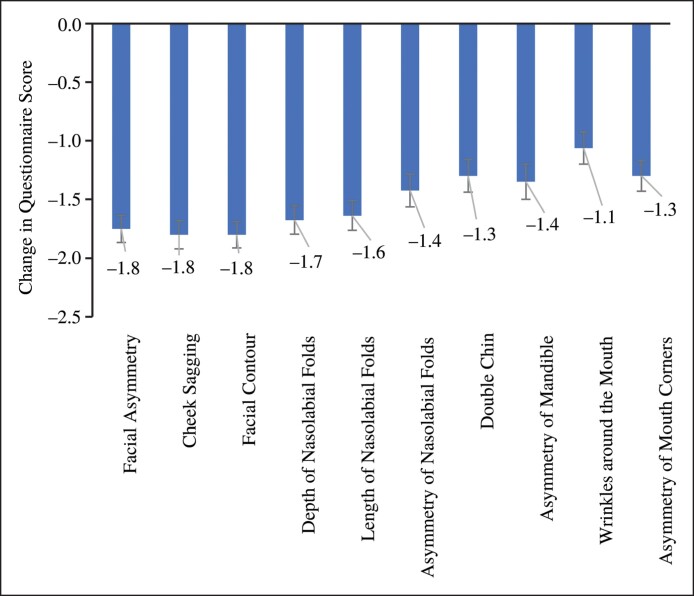
The results of the questionnaire. A significant reduction was observed in all assessed facial characteristics following cosmetic acupuncture. Notably, “facial sagging” and “facial contour” showed a decrease of 1.8 points.

The participant surveys of the 10 cases were analyzed using the Wilcoxon signed-rank test, revealing significant decreases across all items (*P* < .05) following the intervention. Notably, reductions of 1.8 points were observed in facial asymmetry, cheek sagging, and facial contour, reflecting a high level of satisfaction with the treatment (Figure 7, Supplemental Table 2).

## DISCUSSION

In this study, 8 consecutive sessions of cosmetic acupuncture were involved. MRI-based imaging revealed a reduction in masseter muscle volume and alterations in facial contour shape, suggesting the aesthetic benefits of this treatment.

MRI allows a detailed observation of subcutaneous structures because of its high tissue contrast, although it is susceptible to motion artifacts that can affect image quality.^11^ To mitigate this, participants were instructed to avoid jaw movement and minimize saliva swallowing during imaging. Additionally, jaw muscle shape may change because of clenching;^12^ therefore, participants were asked to relax and keep their eyes closed. Imaging experiments were repeated to optimize quality and prevent distortion. To enhance the accuracy of jaw muscle volume measurement, the following controls were implemented:

1. Data before and after the intervention were collected 4 weeks apart.
2. Measurements were conducted randomly to minimize preintervention influence.
3. Two independent assessors measured the jaw muscles 3 times each without access to each other's results, ensuring accuracy across axial, sagittal, and coronal views.

Sato et al^10^ reported decreases in wrinkles and sagging and improvement in skin conditions after 8 treatment sessions. Similarly, Ogino et al^9^ demonstrated that continuous stimulation resulted in changes in the masseter muscle area, whereas Yamazaki et al^14^ noted that acupuncture treatment effectively relieved fatigue after 8 sessions. In contrast, Barrett^1^ observed sustained effects of cosmetic acupuncture after 10 sessions, and Emami Razavi et al^15^ recommended 10 to 12 sessions for achieving optimal results. Through these findings, the authors highlight a difference in the ideal number of sessions between Japanese and foreign patients, yet continuous acupuncture stimulation is considered beneficial in both cases. Given that in our study, only Japanese participants were involved, we adopted an 8-session intervention, in line with previous research conducted in Japan.

Moreover, Shimada et al^16^ found that 86% of participants experienced an immediate facial lift, with 74% maintaining improvements 3 days after treatment. This evidence further supports our decision to standardize MRI 3 days after the final session in our study, ensuring accurate assessment of sustained effects.

In this study, 90% of the participants showed a decrease in jaw muscle volume, with reductions ranging from 4.0% to 13.4% and an average decrease of 7.37%. A statistically significant reduction in muscle volume was confirmed, demonstrating that MRI can objectively measure changes in facial contours. This is a departure from previous studies in which the focus was on superficial skin changes and subjective assessments.

However, 1 participant showed an increase in jaw muscle volume. Guruprasad et al attribute jaw muscle hypertrophy to factors such as teeth grinding, clenching, unilateral chewing, TMJ disorders, malocclusion, and psychological stress.^12^ These factors may have influenced the participant's results, suggesting that acupuncture may not always reduce muscle volume. Because in this study, we did not assess teeth grinding or clenching, we could not perform a further analysis of these factors.

We also evaluated the impact of weight changes on masseter muscle volume, finding no significant effect (Supplemental Table 3). Future research should focus on increasing the number of participants and exploring the relationship between weight and muscle volume.

Facial changes are greatly influenced by aging, with sagging becoming more pronounced because of gravity. Age-related changes in bone and soft tissue can also increase asymmetry.^13^ In the participant questionnaire, significant improvements were observed in all categories, particularly in facial sagging, contours, and asymmetry. The MRI-detected reduction in jaw muscle volume likely contributed to the participants’ psychological satisfaction, as reflected in improved questionnaire scores.

Authors of previous studies on cosmetic acupuncture have shown an increase in blood flow and hydration after a single session, with reductions in wrinkles and sagging and improvement in skin conditions noted after 8 treatments.^2,3,10^ Continuous stimulation has been shown to change jaw muscle area,^16^ and fatigue recovery effects have been observed after 8 sessions.^14^ Barrett and Emami Razavi recommended 10 to 12 sessions for sustained effects,^1,15^ although the optimal frequency may vary between populations. In this study, in which the focus was on Japanese participants, we adopted an 8-session protocol based on previous Japanese studies.

Shimada et al found that 86% of participants perceived an immediate lifting effect, with 74% retaining it 3 days posttreatment. Accordingly, imaging in this study was standardized to 3 days after the final session.^16^

In cases of facial swelling, the acupuncture point ST7, located below the masseter muscle, is commonly used. ST7 is associated with a venous plexus that promotes metabolism through increased blood flow.^17^ Sonehara et al reported that sagging is a primary concern among females,^18^ and acupuncture points ST5 and ST6, located at the masseter muscle attachment sites, are effective for addressing these concerns.^19^

These acupuncture points are also utilized in the treatment of TMJ disorder. Katsuhisa et al emphasized the importance of stabilizing muscle tension in the masticatory muscles for proper jaw function.^20^ Acupuncture at these points is effective in relieving tenderness and nodules associated with muscle tension. Zhang et al discussed acupuncture's role in pain and inflammation relief, noting that it activates α7 nicotinic acetylcholine receptors (α7nAchR) in lung tissues, exerting an antiferroptotic effect, particularly relevant in acute respiratory distress syndrome.^21^

Electroacupuncture is promising for postoperative care, particularly in managing nausea and vomiting. Larson et al found that combining pharmacological prophylaxis with electroacupuncture significantly reduces nausea postsurgery, highlighting its potential as an adjunct therapy.^22^

Botulinum toxin Type A injections are commonly used to reduce masseter muscle hypertrophy and contour the face. Hypertrophy occurs when acetylcholine is released at the neuromuscular junction, triggering muscle contraction. Botulinum toxin inhibits acetylcholine release, reducing muscle hypertrophy.^6^ The injection sites for botulinum toxin are near acupuncture points ST5 and ST6, innervated by the mandibular nerve. Lora et al noted that botulinum toxin significantly reduces substance *P* release, alleviating sensitivity to inflammatory reactions induced by TMJ arthritis.^23^

These mechanisms suggest that botulinum toxin reduces masseter muscle hypertrophy and alleviates pain. DE LA Torre Canales et al found no significant difference in pain reduction between botulinum toxin injections and acupuncture for myofascial TMJ pain,^24^ suggesting that acupuncture may also reduce masseter muscle volume. In this study, pain was found to be minimal, and significant reductions in masseter muscle volume were observed, likely because of improved muscle tension from acupuncture.

The acupuncture points used in this study are the same as those used in TMJ disorder treatment. The influence of patient age and sex should also be considered. In this study, the authors included females with an average age of 50.3 ± 6.45 years, close to the onset age of TMJ disorders. Robinson et al reported a higher incidence of TMJ disorders in females aged 20 to 35 years, with the highest average onset age being 54.5 years.^25^ The higher incidence in females may be because of estrogen depletion during menopause, leading to increased reactive oxygen species and decreased bone mass, affecting the mandible.^26^

Nakamura et al suggested that jawbone changes because of environmental factors may play a role in TMJ disorders in younger individuals, whereas factors in older individuals may include periodontal disease, tooth wear, and prosthetic treatment.^27^ Although the purpose of improving facial appearance may vary with age, TMJ disorders may still affect changes in jaw appearance.

In summary, cosmetic acupuncture, TMJ treatment, and botulinum toxin treatment share insertion sites, suggesting that masseter muscle volume reduction may result from muscle tension relaxation. Although there may be differences in pain presence between these treatments, similarities in insertion sites and patient backgrounds suggest that the reduction in masseter muscle volume observed in 9 participants may be because of muscle tension relaxation (Supplemental Table 4).

### Limitations

In this study, the authors found a few limitations that need to be acknowledged. We included 10 female participants aged 43 to 64 years, with an average age of 50.3 ± 6.45 years. Researchers of future studies should increase participant numbers and include both genders. Further data analysis and specific muscle assessments are needed. MRI may introduce distortions, although efforts were made to minimize them in this study. Imaging before and after the intervention used the same device and sequence, allowing for relative change in evaluation; therefore, further verification of accurate volume measurement is needed. In this study, the authors did not assess lifestyle factors such as diet and teeth grinding, which should be addressed in future research. Follow-up was limited to 3 days postintervention; thus, a longer follow-up is necessary to assess effect persistence. Randomized controlled trials should be considered to establish causality.

In this study, the authors focused on changes in masseter muscle volume following acupuncture stimulation. However, medium- and long-term observations were not included, making the assessment of the duration of cosmetic acupuncture effects difficult. Future researchers should include protocols that allow for these observations and prioritize assessments by independent evaluators, extending to follow-up evaluations to objectively and scientifically evaluate the effects of cosmetic acupuncture.

## CONCLUSIONS

Acupuncture stimulation at acupoints around the masseter muscle can reduce the muscle volume because of muscle relaxation. The improvement in subjective evaluation, as well as changes and decreases in masseter muscle shape, enables the attainment of facial contour aesthetics, contributing to evidence in support of cosmetic acupuncture.

## Supplemental Material

This article contains supplemental material located online at https://doi.org/10.1093/asjof/ojae109.

## Supplementary Material

ojae109_Supplementary_Data
