# Supplementary material for: Effect of Facial Acupuncture Stimulation: MRI-Based Masseter Muscle Volume Analysis and Questionnaire Evaluation
Source: Aesthet Surg J Open Forum. 2024 Nov 10;6:ojae109. doi: 10.1093/asjof/ojae109 (PMC11852262; doi:10.1093/asjof/ojae109)
Supplement: ojae109_Supplementary_Data [file ojae109_Supplementary_Data.zip › Supplementary_Table_3.docx]

**Supplementary Table 3. Weight change in participants and reduction rate of masseter muscle volume**

| **Participant** |  | **Pre-intervention weight (kg)** | **Post-intervention weight (kg)** | **Reduction rate of masseter muscle (%)** |
| --- | --- | --- | --- | --- |
| 1 |  | 53.3 | 53.3 | -2.65 |
| 2 |  | 49 | 49.9 | 1.88 |
| 3 |  | 54 | 53.1 | 3.56 |
| 4 |  | 71 | 69.7 | 1.47 |
| 5 |  | 42.2 | 42 | 6.2 |
| 6 |  | 49.4 | 49.5 | 4.65 |
| 7 |  | 47.5 | 48.2 | 2.17 |
| 8 |  | 50.7 | 49.5 | 2.53 |
| 9 |  | 58 | 58.3 | 4.83 |
| 10 |  | 69 | 58.7 | 4.58 |
| Average |  | 54.4 | 53.2 | 2.92 |
