# Supplementary material for: Effect of Facial Acupuncture Stimulation: MRI-Based Masseter Muscle Volume Analysis and Questionnaire Evaluation
Source: Aesthet Surg J Open Forum. 2024 Nov 10;6:ojae109. doi: 10.1093/asjof/ojae109 (PMC11852262; doi:10.1093/asjof/ojae109)
Supplement: ojae109_Supplementary_Data [file ojae109_Supplementary_Data.zip › Supplementary_Table_4.docx]

**Supplementary Table 4. Effects on the masseter muscle**

| **Intervention** | **Pain relief** | **Muscle relaxation** | **Outcome** | **Target site** |
| --- | --- | --- | --- | --- |
| Cosmetic Acupuncture | -- | Effective | Subjective Effect (Sagging) | Masseter Muscle |
| Reduction in Masseter Muscle Volume | -- | Effective | Reduction in Masseter Muscle | Masseter Muscle |
| Acupuncture for TMJ Syndrome | Effective | Effective | Improvement in Jaw Opening Disorder | Masseter Muscle |
| Botox® treatment | Effective | Effective | Reduction in Masseter Muscle | Masseter Muscle |
