# Supplementary material for: Effect of Facial Acupuncture Stimulation: MRI-Based Masseter Muscle Volume Analysis and Questionnaire Evaluation
Source: Aesthet Surg J Open Forum. 2024 Nov 10;6:ojae109. doi: 10.1093/asjof/ojae109 (PMC11852262; doi:10.1093/asjof/ojae109)
Supplement: ojae109_Supplementary_Data [file ojae109_Supplementary_Data.zip › Supplementary_Table_1.docx]

**Supplementary Table 1. Masseter muscle measurement analysis: standard deviations, mean values, and p-values for paired t-tests**

| **Subject** | **O,M Before standard deviation(cm^3^)** | **I,M Before standard deviation(cm^3^)** | **O,M Aefore standard deviation(cm^3^)** | **I,M Aefore standard deviation(cm^3^)** |
| --- | --- | --- | --- | --- |
| 1 | 1.1491 | 1.9047 | 0.8920 | 2.4983 |
| 2 | 1.2725 | 1.2726 | 1.9712 | 0.6200 |
| 3 | 0.8533 | 0.7407 | 1.5160 | 0.3755 |
| 4 | 0.2603 | 0.0603 | 0.6888 | 0.0231 |
| 5 | 0.4895 | 0.8688 | 0.3356 | 1.0830 |
| 6 | 1.2407 | 1.0209 | 0.6374 | 1.3619 |
| 7 | 0.3585 | 0.6798 | 0.0802 | 0.9950 |
| 8 | 0.2914 | 0.3027 | 0.5680 | 0.7801 |
| 9 | 0.3842 | 0.6471 | 0.2862 | 1.0798 |
| 10 | 1.1399 | 0.9341 | 1.0961 | 0.8190 |
| average | 0.7440 | 0.8432 | 0.8071 | 0.9636 |
| Lilliefors test p-value | 0.1631 | 0.5 | 0.4578 | 0.1456 |
| Paired t-test p-value | 0.355 | | 0.6246 | |
