# Supplementary material for: Effect of Facial Acupuncture Stimulation: MRI-Based Masseter Muscle Volume Analysis and Questionnaire Evaluation
Source: Aesthet Surg J Open Forum. 2024 Nov 10;6:ojae109. doi: 10.1093/asjof/ojae109 (PMC11852262; doi:10.1093/asjof/ojae109)
Supplement: ojae109_Supplementary_Data [file ojae109_Supplementary_Data.zip › Supplementary_Table_2.docx]

**Supplementary Table 2. Average scores before and after intervention for the 10 cases based on participant surveys**

| **Question** | **Facial asymmetry** | **Cheek sagging** | **Facial contour** | **Depth of nasolabial folds** | **Length of nasolabial folds** | **Asymmetry of nasolabial folds** | **Double chin** | **Asymmetry of the mandible** | **Wrinkles around the mouth** | **Asymmetry in mouth corners** |
| --- | --- | --- | --- | --- | --- | --- | --- | --- | --- | --- |
| Before | 3.46 | 3.63 | 3.5 | 3.43 | 3.29 | 3.13 | 2.98 | 2.83 | 2.56 | 2.95 |
| After | 1.71 | 1.83 | 1.7 | 1.75 | 1.65 | 1.7 | 1.68 | 1.48 | 1.5 | 1.65 |
